# Human Alpha-1 Antitrypsin Attenuates ENaC and MARCKS and Lowers Blood Pressure in Hypertensive Diabetic db/db Mice

**DOI:** 10.3390/biom13010066

**Published:** 2022-12-29

**Authors:** Carlos I. Lugo, Lauren P. Liu, Niharika Bala, Angelica G. Morales, Mohammed F. Gholam, Julia C. Abchee, Nasseem Elmoujahid, Ahmed Samir Elshikha, Rigena Avdiaj, Louis A. Searcy, Nancy D. Denslow, Sihong Song, Abdel A. Alli

**Affiliations:** 1 Department of Physiology and Aging, University of Florida College of Medicine, Gainesville, FL 32610, USA; 2 Department of Basic Medical Sciences, College of Medicine, King Saud bin Abdulaziz University for Health Sciences, Jeddah 21423, Saudi Arabia; 3 Department of Pathology, Immunology and Laboratory Medicine, University of Florida College of Medicine, Gainesville, FL 32610, USA; 4 Department of Pharmaceutics, University of Florida College of Pharmacy, Gainesville, FL 32610, USA; 5 Department of Physiological Sciences and Center for Environmental and Human Toxicology, University of Florida College of Veterinary Medicine, Gainesville, FL 32610, USA; 6 Department of Medicine Division of Nephrology, Hypertension, and Renal Transplantation, University of Florida College of Medicine, Gainesville, FL 32610, USA

**Keywords:** MARCKS, AAT, ENaC, kidney, hypertension, diabetes

## Abstract

Hypertension may develop before or after the onset of diabetes and it is known to increase the risk of developing diabetic nephropathy. Alpha-1 antitrypsin (AAT) is a multi-functional protein with beneficial effects in various diseases but its role in reducing blood pressure in the diabetic kidney has not been thoroughly studied. Like blood pressure, epithelial sodium channels (ENaC) and its adaptor protein myristoylated alanine-rich C-kinase substrate (MARCKS) are regulated by circadian rhythms. Our hypothesis is that administration of human AAT (hAAT) reduces blood pressure in hypertensive diabetic mice by attenuating membrane expression of ENaC and its association with the actin cytoskeleton. First, we show hAAT administration results in reduced blood pressure in diabetic db/db mice compared to vehicle treatment in both the inactive and active cycles. Western blotting and immunohistochemistry analyses showed a reduction of ENaC and the actin cytoskeleton protein, MARCKS in the kidneys of diabetic db/db mice treated with hAAT compared to vehicle. hAAT treatment resulted in elevated amounts of extracellular vesicles present in the urine of diabetic db/db mice compared to vehicle treatment both in the inactive and active cycles. Multiple hexosylceramides, among other lipid classes increased in urinary EVs released from hAAT treated hypertensive diabetic mice compared to vehicle treated mice. Taken together, these data suggest hAAT treatment could normalize blood pressure in the diabetic kidney in a mechanism involving attenuation of renal ENaC and MARCKS protein expression and possibly ceramide metabolism to hexosylceramide in kidney cells.

## 1. Introduction

Type 2 diabetes mellitus (T2DM) is a global health problem and it is estimated that the worldwide prevalence of diabetes will increase by 51% by the year 2045 [1]. Excessive glucose in the blood has been shown to increase the risk for cardiovascular disease (CVD) in diabetics through multiple mechanisms such as endothelial dysfunction, insulin resistance, and the toxic effects that glucose has on the vasculature [1]. Hypertension can be categorized as pre-hypertensive (130–139 mmHg systolic and 85–89 mmHg diastolic), stage 1 hypertension (140–159 systolic and 90–99 mmHg diastolic), or stage 2 hypertension (160 mmHg or greater systolic and 100 mmHg or greater diastolic) [2]. There is accumulating evidence that diabetes and hypertension are usually associated with each other [3]. Hypertensive diabetics incur a significantly higher risk of cardiovascular disease, end stage kidney disease, and mortality compared to nondiabetic and normotensive diabetics [3]. Reducing the blood pressure of hypertensive diabetics to a healthy level significantly lowers the risk of cardiovascular disease [3] and renal insufficiency in diabetic nephropathy [4].

Long term control of blood pressure is mediated by the renin angiotensin aldosterone system (RAAS). Aldosterone upregulates epithelial sodium channels (ENaC) in the aldosterone sensitive distal nephron [5]. Aldosterone also induces vasoconstriction, increases the density of the Na/K ATPase in the basolateral membrane and stimulates ATP production. Furthermore, studies have demonstrated that ANGII and aldosterone upregulate ENaC in the collecting duct [6]. ENaC activity is increased when aldosterone binds to the mineralocorticoid receptor [7]. Increasing ENaC protein expression and activity leads to sodium retention and a subsequent increase in blood pressure [8]. Increasing plasma osmolality results in the secretion of antidiuretic hormone (ADH) from the posterior pituitary leading to the translocation of aquaporins 2 (AQP2) to the apical membrane of collecting duct cells. AQP2 makes the collecting duct permeable to water increasing water reabsorption [9]. Once plasma osmolality or blood pressure returns to normal, atrial natriuretic peptide (ANP) is secreted to counteract the effects of RAAS. ANP decreases renin production [10], decreases ANGII and aldosterone [11], induces vasodilation [12], stimulates natriuresis/diuresis to protect the cardiovascular system from pressure and volume overload [13], and inhibits ENaC activity in the kidney [14].

ENaC is regulated by phospholipid phosphates, including phosphatidylinositol 4,5-bisphosphate (PIP2) [15]. PIP2 binds to the β and 𝛾 subunits of ENaC at the amino terminal domain and maintains the channel in an open state [15,16,17]. The myristoylated alanine rich c-kinase substrate (MARCKS) plays a key role in regulating PIP2 availability [16]. PIP2 increases ENaC activity by interacting with the amino terminal domain of the β and 𝛾 subunits of ENaC [16]. The interaction between MARCKS and ENaC is negatively regulated by protein kinase C (PKC) dependent phosphorylation [16]. Furthermore, ENaC activity is regulated by several tryptic proteases such as trypsin [18], furin [19,20], cathepsin B [21,22], cathepsin S [23], prostasin [19,24], and kallikrein [25].

In addition, circadian rhythms play an important role in the physiological control of renal function and blood pressure. There are four gene families that make up the circadian clock mechanism: Cryotochrome (Cry), Period (Per), brain and muscle Arnt-like protein (Bmal), and Clock [26]. Previous studies have demonstrated that Per1 regulates ENaC activity [26,27]. Regulation of ENaC by Per1 occurs at the transcriptional and functional level [26]. Knocking out Per1 is associated with a decrease in ⍺ENaC activity and expression in the kidney [28]. Furthermore, a study using Clock^−/−^ mice showed that aldosterone secretion and urinary excretion of sodium and potassium no longer adhered to a circadian rhythm in these mice [29].

Alpha-1 antitrypsin (AAT) is produced mainly by the liver and secreted into the circulation and it plays an essential role in protecting the lung from the enzyme neutrophil elastase [30]. Studies have demonstrated that AAT also has anti-inflammatory properties [30]. Additionally, AAT is an endogenous inhibitor of renin [31,32], which plays an important role in the RAAS cascade. In a healthy human, approximately 34 mg of AAT per kilogram of body mass is secreted into the circulation by the liver each day and AAT has a half-life of 3 to 5 days [33]. AAT levels in the circulation can increase significantly during an acute inflammatory response [30]. Research in mouse models demonstrated therapeutic potential for AAT in various diseases including arthritis [34], osteoporosis [35], lupus [36], and type 1 diabetes [37,38]. Previous studies have demonstrated that AAT protected beta cells from apoptosis [39].

Excessive sodium retention and hypertension are caused by excessive protein expression and activity of ENaC and presumably its adaptor protein MARCKS and proteases that regulate them. Liu et al. demonstrated that cathepsin B activity was significantly decreased in transgenic mice that overexpressed hAAT [40]. Therefore, in this study we tested the hypothesis that administering a physiological dose of hAAT to hypertensive diabetic db/db mice will alleviate hypertension by decreasing ENaC and MARCKS protein expression and activity. We also investigated the effects that hAAT has on electrolyte and blood pressure regulation in the inactive and active cycle of hypertensive diabetic db/db mice.

## 2. Materials and Methods

### 2.1. Experimental Design

**Figure 1:**
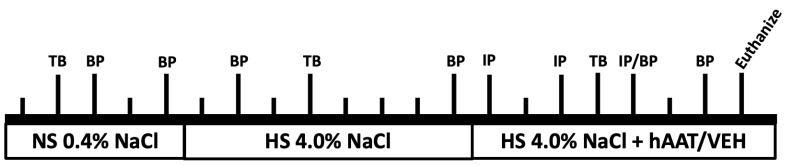
Schematic of study design. Each vertical line represents one day. Tail bleeds (TB), Blood pressure (BP), intraperitoneal (IP) injection of either hAAT or vehicle. There were 3 phases: normal salt (NS), high salt (HS), and high salt plus treatment (hAAT) or vehicle (VEH).

Figure 1. Schematic of study design.

### 2.2. Animals

Male (N = 8) and female (N = 8) diabetic db/db mice (BKS.Cg-Dock7^+/+^ Lepr/J; Stock No: 000642) were purchased from the Jackson Laboratory (Bar Harbor, ME, USA), the mice were 10 weeks old at the start of the study. All our animal studies were performed under an approved University of Florida Institutional Animal Care and Use Committee protocol and these studies were in compliance with the National Institutes of Health “Guide for the Care and Use of Laboratory Animals”.

### 2.3. Blood Pressure Measurements

Baseline blood pressure measurements were performed using the tail-cuff method (IITC MRBP System from Life Science Inc.; Woodland Hills, CA, USA) in the AM (8 AM–10 AM) and in the PM (8 PM–10 PM). This blood pressure machine specifically designed for mice and rats was used to analyze the data and systolic blood pressure was defined according to the AHA. Blood pressure was taken on the third and fifth days of the normal salt diet of 0.40% NaCl (Teklad, Envigo, Indianapolis, IN, USA) (Figure 1). To induce hypertension in adult diabetic db/db mice, we fed them a high salt diet of 4.0% NaCl (Teklad, Envigo) for 8 days. Blood pressure was measured on the second and eighth days of the high salt diet (Figure 1). The mice were maintained on the high salt diet and were given hAAT or vehicle every other day. The blood pressure was taken on the fifth and seventh days of the treatment phase with hAAT or vehicle.

### 2.4. Human AAT Injections and Detection

Hypertensive diabetic db/db mice were given either vehicle or clinical grade hAAT (Prolastin^®^C, Grifols, 2 mg per mouse, 0.035 kg body weight) via intraperitoneal (IP) injection. The vehicle group consisted of 4 male and 4 female salt-sensitive hypertensive db/db mice. Similarly, the hAAT treated group consisted of 4 male and 4 female salt-sensitive hypertensive db/db mice. The mice received one injection at 11 AM, every other day, for a total of three injections. For the detection of hAAT in the circulation, mouse serum samples were run on an hAAT specific ELISA as previously described by Elshikha et al. [41].

### 2.5. Tissue Processing

Fifty milligram sections of the right kidney cut longitudinally was homogenized in 500 µL of tissue protein extraction reagent (TPER) (Thermo Fisher Scientific; Waltham, MA, USA) using an Omni TH homogenizer (Warrenton, VA, USA). The lysates were incubated for 20 min on ice and vortexed every 5 min. After incubation the tissue lysates were centrifuged at 13,000 rpm for 10 min in a micromax benchtop centrifuge (Thermo IEC). The supernatant was obtained and subjected to ultracentrifugation in a optima L-90K ultracentrifuge (Beckman Coulter; Schaumburg, IL, USA) for 30 min at 34,000 rpm at 4 °C using a SW55.1 rotor (Beckman Coulter). The supernatant was collected and represented the soluble fraction. Next, 250 µL of TPER was used to reconstitute the pellet before being sonicated for two 5 s intervals and this constituted the membrane fraction. A BCA assay (Thermo Fisher Scientific) was performed to determine the protein concentration of the soluble and membrane fractions.

### 2.6. SDS-PAGE and Western Blotting

Eighty micrograms of soluble or membrane fraction kidney cortex tissue lysates were loaded into 12-well polyacrylamide gels (Thermo Fisher Scientific). The proteins were resolved on a Criterion electrophoresis system (BioRad; Hercule, CA, USA) at 200 V for an hour. The proteins were electrophoretically transferred to nitrocellulose membranes (Thermo Fisher Scientific) using a Criterion transfer system (BioRad) in Towbin buffer (25 mM Tris, 192 mM glycine, 20% methanol) at 100 V for two hours. The membranes were blocked using a 5% non-fat milk 1×Tris Buffered Saline (TBS) solution for one hour. The membranes were washed twice with 1×TBS solution, and then incubated for 8–12 h in primary antibodies (ENaC alpha 59 [16]), MARCKS (abcam, ab72459), aquaporin-2 (abcam, ab230170), NPRC [42], cathepsin B (Cell Signaling Tech, 3383, Danvers, MA, USA), Kallikrein (Boster Bio, PA2038, Pleasanton, CA, USA), caveolin-1 (Cell Signaling Tech, ab2910), annexin A2 (Cell Signaling Tech, 8235), GAPDH-HRP (Cell Signaling Tech, 3683), and TSG101 (abcam, ab30871) at 4 degrees Celsius. The membranes were washed three times with 1×TBS before being incubated with a 1:3000 dilution of goat anti-rabbit secondary antibody (BioRad) solution prepared in blocking buffer. After a series of 4 washes with 1×TBS, the membranes were incubated with ECL reagent (BioRad) for six minutes and then imaged using a Bio-Rad imager.

### 2.7. Cathepsin B and S Activity Assays

Cathepsin B and S activities in the soluble fractions of kidney cortex tissue lysates collected from each mouse was measured using the Cathepsin B and S Activity Assay Kits (Abcam) according to manufacturer instructions.

### 2.8. Immunohistochemistry

The left kidney from each mouse was cut longitudinally, formalin-fixed for 24 h, washed with 1× Phosphate Buffered Saline (PBS) (137 mM NaCl, 2.7 mM KCl, 10 mM Na_2_HPO_4_, 1.8 mM KH_2_PO_4_) before placing in 70% ethanol, and then cut into 4μm sections for immunohistochemistry. The paraffin-embedded kidney tissue sections underwent 2 exchanges of xylene (Fisher Scientific; Pittsburgh, PA, USA), followed by 2 exchanges of 100% ethanol, 1 exchange of 95% ethanol, 1 exchange of 70% ethanol, 1 exchange of 50% ethanol and then 1 exchange of type 1 water for 3 min intervals. The slides were then placed in boiling citrate buffer for 20 min (Vector laboratories, Inc.; Burlingame, CA, USA), followed by a 3 min wash in type 1 water. Afterwards, the slides were then placed in 1×PBS (Corning; Manassas, VA, USA) for 5 min. The tissues were then blocked with 2.5% normal horse serum (Vector laboratories, Inc.) and then incubated for 20 min in a humidified chamber at room temperature. Next, 200 µL of primary antibody (1:250 dilution was used for both ENaC alpha mouse monoclonal antibody (StressMarq, catalogue# SMC-242D) and MARCKS rabbit polyclonal antibody (abcam, catalogue# 12-548-B) was added per tissue section and incubated for 60 min in a humidified chamber. The rabbit polyclonal anti-MARCKS antibody was added first and incubated with the tissue for 60-min. After a wash with 1×PBS the tissue was incubated for 60 min with the mouse monoclonal ENaC alpha antibody in a humidified chamber. The slides were then washed in 1×PBS for a total of 3 exchanges for 2 min. One drop of VectaFluor Duet Reagent (Vector laboratories, Inc.) was added to each tissue and incubated for 30 min at room temperature in a humidified chamber. Three 2 min washes were performed using 1×PBS, followed by one drop of Vectashield anti-fade mounting media (Vector laboratories, Inc.) before applying a 22X22-1 glass coverslip (Fisher Scientific). The slides were imaged for fluorescence using a Nikon TE microscope with a 40× objective.

### 2.9. Measurement of Urine Osmolality

Urine osmolality was measured using a 2430 Multi-OSMETTE Auto-Sampling Turntable Osmometer (Precision Systems Inc.; Natick, MA, USA). Urine samples were vortexed for 5 s and 30 µL of urine was aliquoted into the tubes and analyzed.

### 2.10. Measurements of Urine Electrolytes

The concentration of urinary sodium was measured using a AU5822 clinical chemistry analyzer (Beckman Coulter; Brea, CA, USA). The urine was centrifuged at 13,000 rpm for 6 min in a micromax benchtop centrifuge (Thermo IEC) before being analyzed. The urinary concentration of potassium and chloride was measured using a SmartLyte Electrolyte ISE Analyzer (Diamond Diagnostics, Holliston, MA, USA). The urine was centrifuged at 13,000 rpm for 6 min in a micromax benchtop centrifuge (Thermo IEC), the supernatant was aliquoted and diluted in urine diluent (Diamond Diagnostics) using a 1:3 dilution. The samples were vortexed for 3 s before being analyzed.

### 2.11. Blood Glucose Measurements

Five µL of blood collected during the normal salt phase and the treatment phase was analyzed using a CVS Health Advanced Glucose Meter (CVS; Woonsocket, RI, USA) according to the manufacturer’s instructions.

### 2.12. Urinary Extracellular Vesicle Isolation and Nanoparticle Tracking Analysis

EVs were isolated from 10 mL’s of urine from diabetic db/db mice collected during the active cycle (AM collection) or inactive cycle (PM collection) while the mice were in metabolic cages. The urine samples were centrifuged at 1000× *g* for 15 min at 4 °C before being filtered through a 0.2 μm rapid-flow Nalgene filter (Thermo Fisher Scientific). The supernatant was then subject to ultracentrifugation at 52,000 rpm for 90 min at 4 °C using a ti-70 fixed-angle rotor (Beckman Coulter, Inc., Brea, CA, USA). The resulting EV pellet was resuspended in 200 μL of ultra-pure 1×PBS (ThermoFisher) and then aliquoted and stored at −80 °C. To measure EV size and concentration, nanoparticle tracking analysis was performed using a NanoSight NS300 (Malvern Panalytical, Malvern, UK) machine equipped with NTA 3.4 Build 3.44 Software (Malvern) as previously described by our group [43].

### 2.13. Transmission Electron Microscope Analysis of Freshly Isolated EVs

A solution of 1×PBS and paraformaldehyde (2% final concentration) was used to reconstitute freshly isolated EV pellets. Formvar-carbon coated grids (Ladd Research Industries; Williston, VT, USA) were incubated with 10 μL of the EV suspensions for 20 min at room temperature for absorption. Next, 100 μL of 1×PBS was aliquoted onto the grids and incubated for 3 min. The grids were then transferred to 50 μL of 1×PBS and 1% glutaraldehyde (Ladd Research Industries) and allowed to incubate for 5 min at room temperature. A series of eight 2 min washes in 50 μL of 1×PBS was then performed. After the washes, the grids were then incubated at room temperature in 50μL of uranyl-oxalate solution, pH 7 prepared from uranyl acetate (Ladd Research Industries) and oxalic acid (Sigma-Aldrich; St. Louis, MO, USA). Afterward, the grids were placed in 50 μL of methyl cellulose-uranyl-acetate and incubated for 10 min on a cold plate. Using a stainless-steel loop, the grids were carefully removed and blotted onto a No.1 Whatman filter paper. The grids were then allowed to air dry for 5 min. A Hitachi H-7600 transmission electron microscope (Hitachi High Technologies America, Inc., Clarksburg, MD, USA) equipped with AMT imaging software (Advanced Microscopy Techniques Corporation, Woburn, MA, USA) was used to view the grids.

### 2.14. Lipidomics

EVs were extracted for lipids using the Bligh and Dyer method [44], as described previously [45]. Briefly, samples were adjusted to equivalent EV number and adjusted to 1 mL using water in a 10-mL glass screw-capped tube. The samples were spiked with an internal standard (EquiSPLASH Lipidomix, Avanti Polar Lipids, Inc., Birmingham, AL, USA), consisting of a mix of 13 deuterated lipids, each at a concentration of 20 ug/mL. Nine hundred µL methanol and 2 mL methylene chloride were added to the mixture and vortexed for 30 s. This was immediately followed by an additional 1 mL water and 0.9 mL methylene chloride; the samples were well mixed and centrifuged at 200× *g* for 10 min. The organic lower phase was carefully collected and dried under a stream of N_2_. The samples were reconstituted in 50 µL methanol and used for LC-MS/MS analysis.

Lipid samples were analyzed using a Nexera X2 ultra-high-performance liquid chromatography instrument (UHPLC, Shimadzu Co., Kyoto, Japan) linked to a QTRAP 6500 mass spectrometer (AB SCIEX, Redwood Shores, CA, USA). Chromatographic separation was performed using a Luna NH2 column (100 × 2 mm column, Phenomenex, Torrence, CA, USA). Lipids were eluted from the column using a binary gradient of dichloromethane:acetonitrile, 7:93 (v,v) for mobile phase A and acetonitrile:water, 50:50 (v,v), pH 8.2, (adjusted with NaOH) for mobile phase B. Both mobile phases contained 2 mM ammonium acetate. The needle rinse was isopropyl alcohol. The flow rate began at 0.2 mL/min and ramped up to 0.7 mL/min within 2 min. Mobile phase B started at 0% and ramped up to 50% within 11 min, then to 70% by min 11.5 and to 100% by min 12.5, and holding at 100% till min 15 and then returning to 0% by 15.1 min and stopping by 17 min. The mass spectrometer acquisition used scheduled multiple reaction monitoring (MRM) in both positive and negative ionization modes, as described previously [45].

The collision energy was varied ranging from 25–50 depending on the lipid species [46]. Declustering potential of the electrospray ionization source was set to 80 in negative mode and 60 in positive mode. The entrance potential was set to 10 and collision cell exit potential was set to 15 in both polarities. The source parameters were curtain gas 45, collision gas 8, temperature 400 °C, ion source gas 1 at 50, and ion source gas 2 at 60 for both polarities. The ion spray voltage was 5500 for positive mode and −4500 for negative mode. Data were acquired using Analyst software (AB SIEX, Framingham, MA, USA, ver. 1.6.2). Surrogate internal standards were assigned based on lipid class and retention time. Data processing was performed in MultiQuant software (AB SCIEX, ver. 3.0.3). The analyte to internal standard peak area ratio was used for lipid quantification. The concentration of lipids was expressed as µM.

### 2.15. Statistical Analysis

To analyze the statistical significance of the data, we performed a Student’s *t*-test if there were only two groups being compared. A One-Way ANOVA followed by Holm–Sidak comparison was performed to compare multiple groups. If gender differences were not significant, we pooled the data for both males and females to compare differences between the two treatment groups. We used a significance level of *p* < 0.05.

## 3. Results

### 3.1. Blood Glucose Levels in Salt Sensitive Hypertensive db/db Mice after hAAT Treatment

First, to verify diabetes, we measured blood glucose at the start of the study and during the treatment phase with hAAT or vehicle. All the mice remained diabetic throughout the study; their blood glucose was greater than the glucose meter’s reportable range which is greater than 600 mg/dL.

### 3.2. Circulating hAAT Levels in Hypertensive Diabetic db/db Mice after hAAT Administration

We collected plasma from the diabetic db/db mice one day after hAAT (2 mg/mouse, every 2 days) or vehicle administration. ELISAs were performed to detect circulating levels of hAAT. As expected, the control group of mice that received vehicle did not show any detectable levels of hAAT in their plasma. Conversely, the hypertensive diabetic db/db mice that were injected with hAAT had an average of 228.9 μg/mL of hAAT with a standard deviation of ±186.6 in the inactive cycle. For the active cycle, the mice had an average of 145.5 μg/mL of hAAT with a standard deviation of ±52.9.

### 3.3. Presence of hAAT in Kidney Cortex Lysates from hAAT Treated Mice Compared to Control Mice

**Figure 2:**
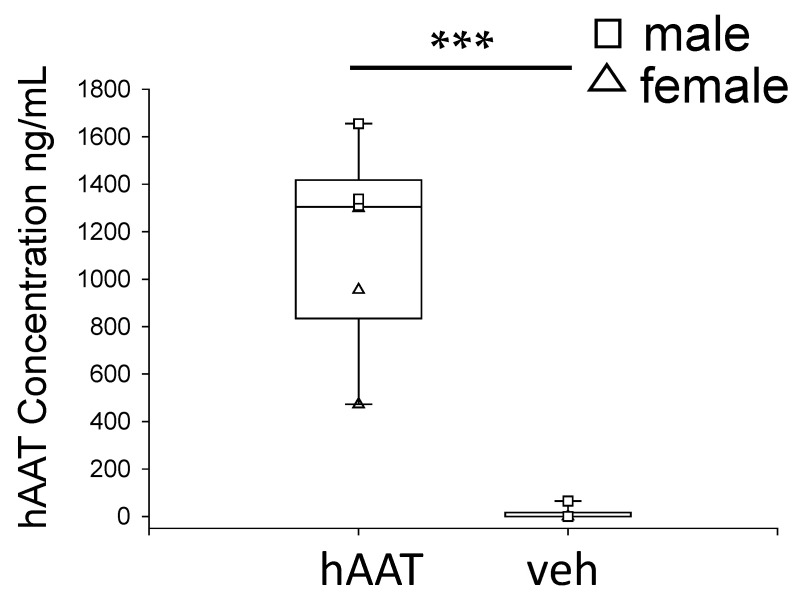
hAAT detection in kidney cortex lysates of db/db mice after hAAT treatment compared to vehicle treatment. The concentration (ng/mL) of hAAT in hypertensive diabetic db/db mice treated with hAAT or vehicle was measured by ELISA. The total volume of kidney lysate used was 15 µL. *** *p* < 0.001.

To determine whether hAAT enters renal epithelial cells, we probed for hAAT in kidney cortex lysates from hAAT treated and vehicle treated db/db mice. As shown in Figure 2, hAAT levels were striking in mice injected with hAAT compared to mice injected with vehicle.

### 3.4. Administration of hAAT Decreases Systolic Blood Pressure in Hypertensive Diabetic db/db Mice

**Figure 3:**
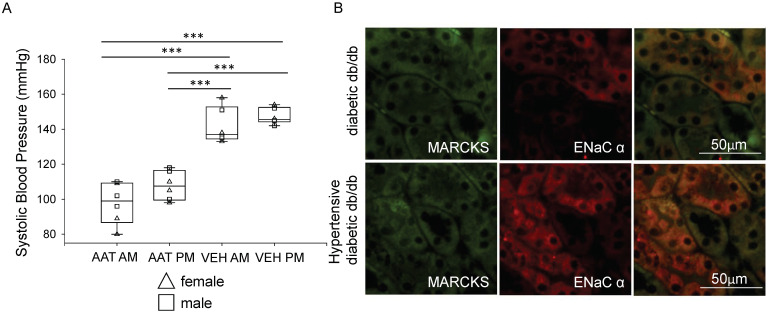
Changes in systolic blood pressure in hypertensive diabetic db/db mice after treatment with vehicle or hAAT. (**A**). Tail cuff systolic blood pressure measurements during the inactive (AM) and active (PM) cycles for female and male hypertensive diabetic db/db mice treated with hAAT and hypertensive diabetic db/db mice given vehicle in which both groups were maintained on a high salt (HS) diet. N = 6 hAAT treated (3 females and 3 males) and N = 6 VEH (3 females and 3 males) mice. These results were analyzed using a One-Way ANOVA followed by a Holm–Sidak comparison. *** represents a *p* < 0.001. (**B**). Representative immunohistochemistry images of MARCKS and ENaC alpha proteins in non-salt loaded diabetic db/db mice and salt-loaded hypertensive diabetic db/db mice (N = 6 mice in each group).

Blood pressure of hypertensive diabetic db/db mice treated with hAAT was significantly lower during the inactive cycle when compared to the hypertensive db/db mice that received vehicle for the active and inactive cycles (Figure 3A). There was also a significant decrease in blood pressure during the active cycle of hAAT treated hypertensive diabetic db/db mice when compared to the blood pressure of hypertensive diabetic db/db mice that received vehicle for both the inactive and active cycles (Figure 3A). The blood pressure also demonstrated a circadian rhythm where the blood pressure was slightly higher during the active cycle compared to the inactive cycle (Figure 3A). We performed immunohistochemistry to demonstrate that ENaC contributes to the development of salt sensitive hypertension. A high salt diet (4.0% NaCl) resulted in a noticeable increase in ENaC alpha protein expression and increased co-localization with MARCKS in the kidneys of db/db mice when compared to kidneys of db/db mice on a normal salt diet (0.4% NaCl) (Figure 3B).

### 3.5. Urinary Osmolality and Electrolytes in Diabetic db/db Mice Treated with hAAT or Vehicle

Next, we investigated whether salt loading alters urine osmolality in diabetic db/db mice, however, there was not a significant difference between the hAAT treated and vehicle treated diabetic db/db mice (Table 1). Additionally, there was no change in urine electrolyte concentration between the hAAT treated and vehicle treated diabetic db/db mice (Table 1).

### 3.6. Effect of hAAT Administration on ENaC Alpha Subunit Protein Expression

**Figure 4:**
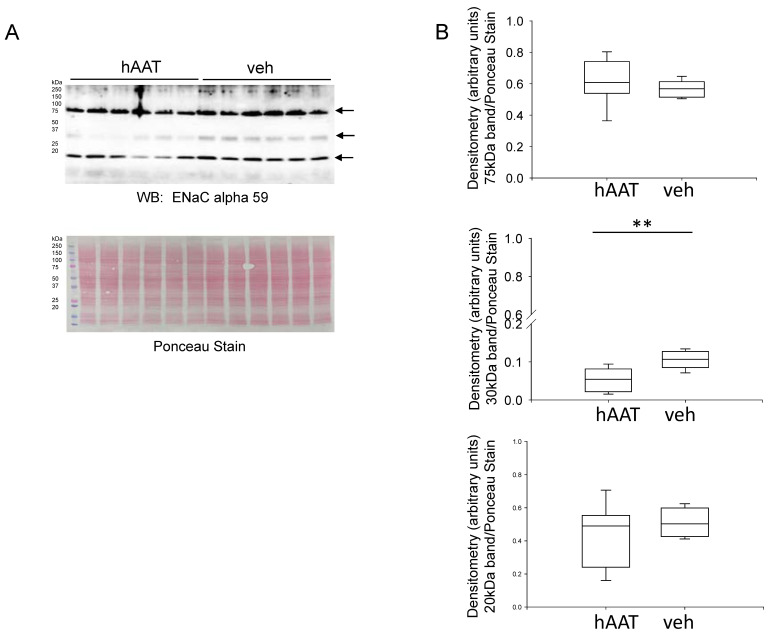
Western blot and densitometric analysis for ENaC alpha. (**A**). Western blot for ENaC alpha subunit protein expression. Eighty micrograms of total protein from kidney cortex lysates from hypertensive diabetic db/db mice treated with either hAAT or vehicle were resolved by SDS-PAGE and then transferred to nitrocellulose membranes which were blotted for ENaC alpha subunit protein expression. (**B**). Image J was used to obtain densitometry values for each band which were normalized to the Ponceau staining. N = 6 mice in each group. ** *p* < 0.01.

Several epithelial transport proteins that contribute to electrolyte balance, urine composition, and blood pressure are expressed in the distal nephron and collecting duct of the kidney. First, we investigated whether hAAT treatment alters protein expression of the alpha subunit of ENaC in the diabetic kidney. As shown in Figure 4, the 30 kDa immunoreactive band corresponding to ENaC alpha was significantly decreased in the kidney cortex lysates from db/db mice treated with hAAT compared to vehicle.

### 3.7. MARCKS Protein Expression Is Reduced in the Diabetic db/db Kidney after Treatment with hAAT

**Figure 5:**
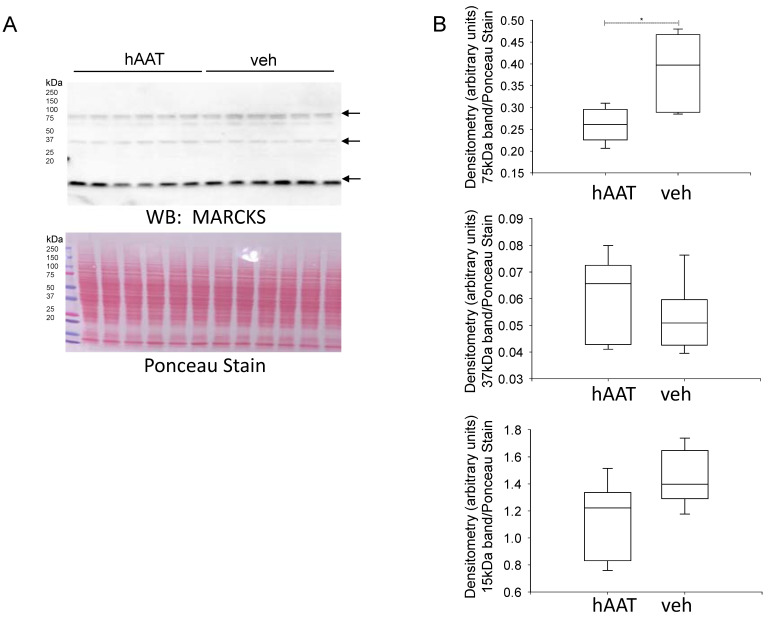
Western blot and densitometric analysis of MARCKS protein expression after hAAT or vehicle treatment. (**A**). Western blot for MARCKS protein expression. Eighty micrograms of total protein from kidney cortex lysates from hypertensive diabetic db/db mice treated with either hAAT or vehicle were resolved by SDS-PAGE and then transferred to nitrocellulose membranes which were blotted for MARCKS protein expression. (**B**). Image J was used to obtain densitometry values for each band which were normalized to the Ponceau staining. N = 6 mice in each group. * *p* < 0.05.

The actin cytoskeleton protein MARCKS plays an essential role in sequestering anionic phospholipids phosphates and stabilizing ENaC in an open conformation at the apical plasma membrane. Like ENaC, MARCKS is subject to proteolysis by a myriad of proteases but its regulation at the protein level has not been investigated in diabetic mice treated with hAAT. Western blot and densitometric analysis showed a significant reduction of a 75 kDa immunoreactive band corresponding to MARCKS protein expression in the soluble fraction of kidney cortex lysates from diabetic db/db mice treated with hAAT compared to vehicle (Figure 5).

### 3.8. hAAT Treatment Attenuates Colocalization of MARCKS and ENaC Alpha Protein in the Diabetic db/db Kidney

**Figure 6:**
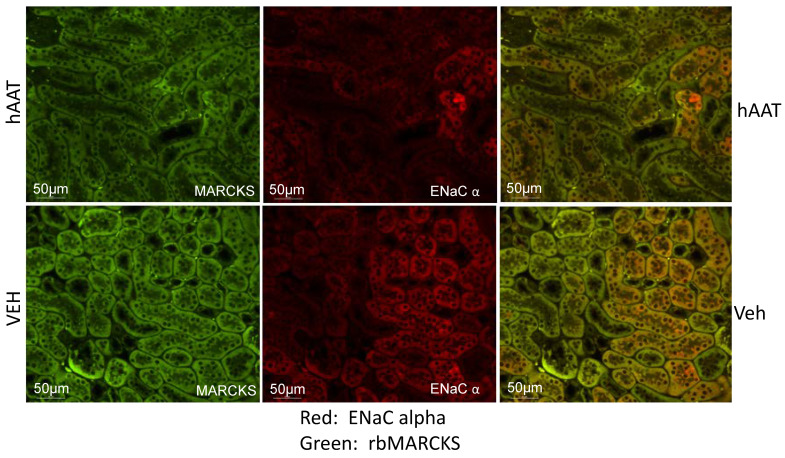
Colocalization of MARCKS and ENaC alpha subunit protein expression in the kidney cortex of hypertensive diabetic db/db mice after treatment with vehicle or hAAT. Representative immunohistochemistry images using a mouse monoclonal antibody against the alpha subunit of ENaC and a rabbit polyclonal antibody against recombinant MARCKS protein were used to investigate colocalization of the two proteins in the hypertensive diabetic db/db kidney. N = 6 mice in each group.

The direct interaction between MARCKS and ENaC is essential for the PIP2 dependent regulation of ENaC. To date, no study has investigated whether hAAT treatment regulates the co-localization and association between MARCKS and ENaC in the diabetic kidney. To corroborate the decreased ENaC alpha subunit and MARCKS protein expression observed by Western blot analysis, we performed immunohistochemistry analysis and further investigated colocalization of MARCKS and ENaC alpha protein expression in the kidneys of diabetic db/db mice. hAAT treatment was shown to dramatically reduce colocalization of MARCKS and ENaC alpha in the kidneys of diabetic db/db mice compared to vehicle treated mice (Figure 6).

### 3.9. hAAT Treatment Does Not Alter Aquaporin-2 Protein Expression in the Diabetic Kidney

**Figure 7:**
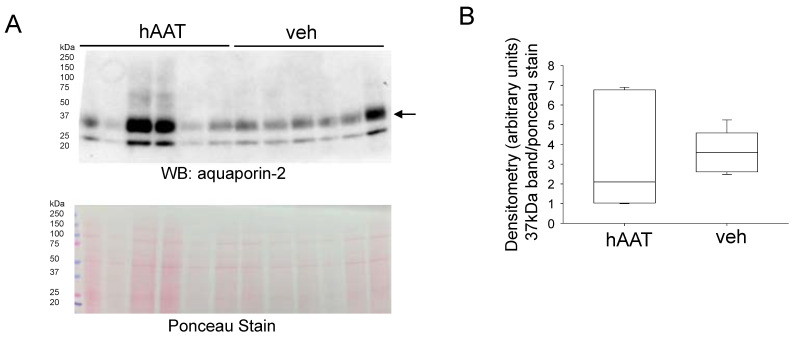
Western blot and densitometric analysis of aquaporin-2 protein expression in the hypertensive diabetic kidney. (**A**). Western blot for aquaporin-2 protein expression. Eighty micrograms of total protein from kidney cortex lysates from hypertensive diabetic db/db mice treated with either hAAT or vehicle were resolved by SDS-PAGE and then transferred to nitrocellulose membranes which were blotted for aquaporin-2 protein expression. (**B**). Image J was used to obtain densitometry values for each band which were normalized to the Ponceau staining. N = 6 mice in each group.

Since other membrane proteins at the luminal membrane of the kidney collecting duct are regulated by the actin cytoskeleton, we also investigated whether hAAT treatment alters their protein expression. First, we investigated whether protein expression of aquaporin-2 in the hypertensive diabetic db/db kidney is regulated by hAAT treatment. Western blot and densitometric analysis did not show an appreciable difference in aquaporin-2 protein expression between the two groups (Figure 7).

### 3.10. hAAT Treatment Does Not Alter NPRC Protein Expression in the Kidney of db/db Mice

**Figure 8:**
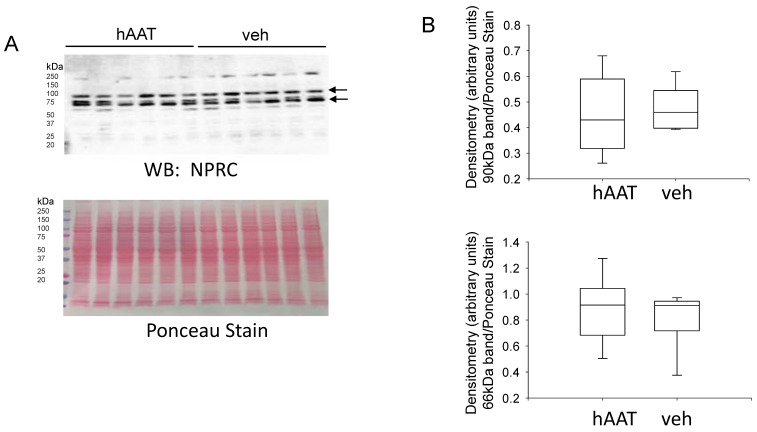
Western blot and densitometric analysis of NPRC protein expression after hAAT or vehicle treatment. (**A**). Western blot for NPRC protein expression. Eighty micrograms of total protein from kidney cortex lysates from hypertensive diabetic db/db mice treated with either hAAT or vehicle were resolved by SDS-PAGE and then transferred to nitrocellulose membranes which were blotted for NPRC protein expression. (**B**). Image J was used to obtain densitometry values for each band which were normalized to the Ponceau staining. N = 6 mice in each group.

NPRC is another protein that is expressed at the luminal membrane of kidney collecting duct cells which express MARCKS and ENaC. NPRC is responsible for clearing natriuretic peptides from the circulation, but numerous studies have shown that this receptor is also coupled to phospholipase C and adenyl cyclase signal transduction. Natriuretic peptides can inhibit ENaC at two levels. First, natriuretic peptides can inhibit ENaC activity in a cGMP dependent manner. Second, natriuretic peptides binding to NPRC can lead to increased hydrolysis of PIP2 by activation of phospholipase C. Here, we investigated whether hAAT alters NPRC protein expression in the diabetic db/db kidney. Western blot analysis and densitometric analysis did not show a change in NPRC protein expression between the two groups (Figure 8).

### 3.11. hAAT Treatment Does Not Alter Cathepsin B or Kidney Kallikrein Protein Expression or Activity in db/db Mice

**Figure 9:**
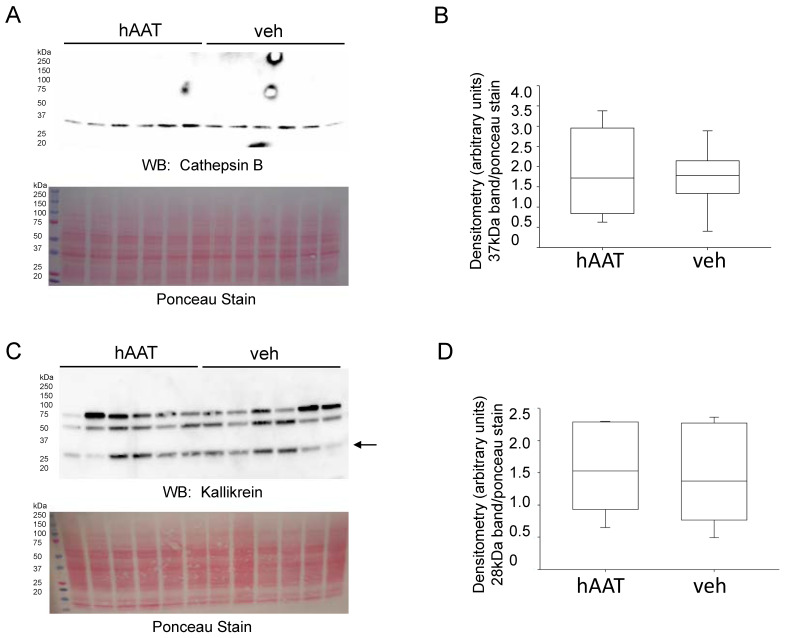
Western blot and densitometric analysis of cathepsin B and kidney kallikrein protein expression after hAAT or vehicle treatment. (**A**). Western blot for cathepsin B protein expression. Eighty micrograms of total protein from kidney cortex lysates from hypertensive diabetic db/db mice treated with either hAAT or vehicle. (**B**). Densitometry values were obtained using Image J for the cathepsin B band in panel A normalized to the Ponceau staining. N = 6 mice in each group. (**C**). Western blot for kallikrein protein expression. Eighty micrograms of total protein from kidney cortex lysates from hypertensive diabetic db/db mice treated with either hAAT or vehicle. (**D**). Densitometry values were obtained using Image J for the kallikrein band in panel C normalized to the Ponceau staining. N = 6 mice in each group.

Since cathepsin B and kallikrein are both known to cleave and activate ENaC we next investigated if hAAT treatment alters cathepsin B expression and activity. Our initial studies showed AAT inhibits cathepsin B activity in vitro. Next, we investigated whether injection of db/db mice with hAAT (2 mg/mL, every 2 days) would alter cathepsin B and kidney kallikrein protein expression and activity. Figure 9 shows there was no appreciable change in either cathepsin B or kidney kallikrein protein expression in diabetic db/db mice injected with hAAT compared to vehicle. Similarly, there was no difference in the activity of either protease present in the plasma of the diabetic db/db mice treated with hAAT compared to vehicle (Table 2).

### 3.12. Characterization of Urinary EVs from db/db Mice Treated with hAAT or Vehicle

**Figure 10:**
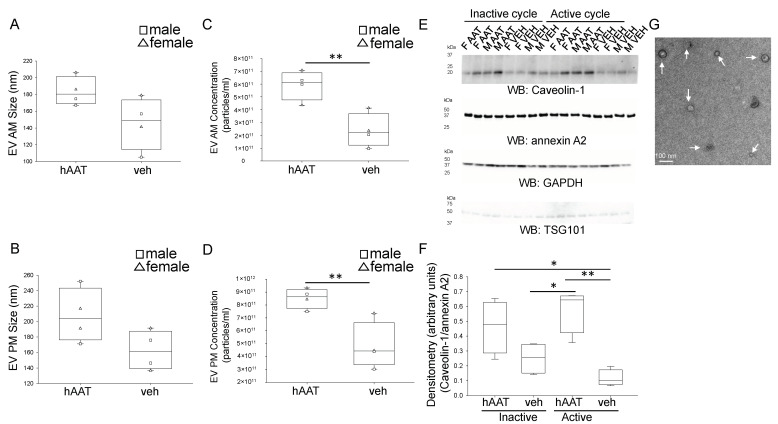
Characterization of urinary EVs from hAAT and vehicle treated db/db mice. (**A**). Summary graph for EV size from the active (AM) cycle. (**B**). Summary graph for EV size from the inactive (PM) cycle. (**C**). Summary graph for EV concentration from the active (AM) cycle. (**D**). Summary graph for EV concentration from the inactive (PM) cycle. (**E**). Western blots for multiple EV markers including caveolin-1, annexin A2, GAPDH, and TSG101. (**F**). Densitometric analysis of the caveolin-1 blot normalized to the Annexin A2 blot. (**G**). Representative TEM image of uEVs from diabetic db/db mice. AM reflects urine collected overnight during the active cycle of the mice and PM reflects urine collected during the day of the inactive cycle of the mice. White arrows indicate individual uEVs. * *p* < 0.05 and ** *p* < 0.01.

Recent studies have shown the packaged cargo of EVs can regulate several cellular mechanisms and membrane proteins including ENaC [39]. To determine whether hAAT treatment affects the amount of EVs released into the urine we isolated urinary EVs from urine samples from the active (AM urine collection) or inactive (PM urine collection) cycle from each mouse. First, we characterized the EVs by nanoparticle tracking analysis to compare EV size and concentration between the groups. Urinary EV size appeared to increase in the inactive and active cycles of hAAT treated diabetic db/db mice (Figure 10A,B). Moreover, the concentration of urinary EVs was greater for the hAAT treated mice compared to the control group in both the inactive and active cycles (Figure 10C,D). Next, we characterized the EVs by Western blot for multiple EV markers (Figure 10E,F). Of these markers, we found the enrichment of caveolin-1 was striking between the hAAT and vehicle treated mice. To confirm the size and purity of the EV preparations, transmission electron microscopy (TEM) was performed. Figure 10G shows a representative TEM image of urinary EVs from diabetic db/db mice.

### 3.13. hAAT Treatment Increases Hexosylceramides in uEVs from Diabetic db/db Mice

**Figure 11:**
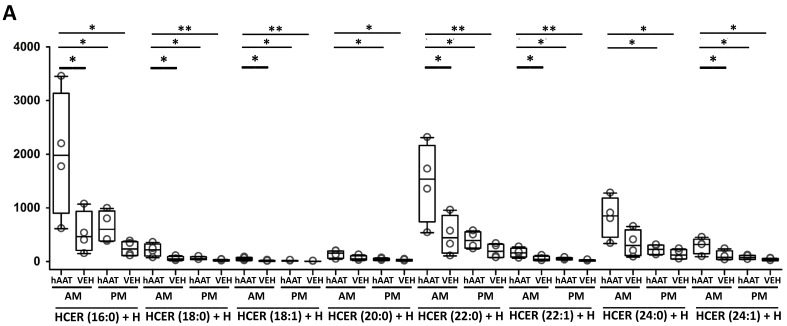

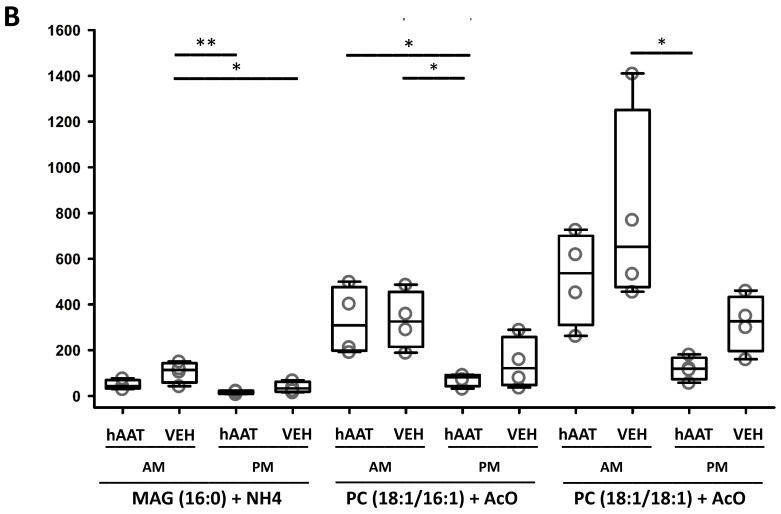

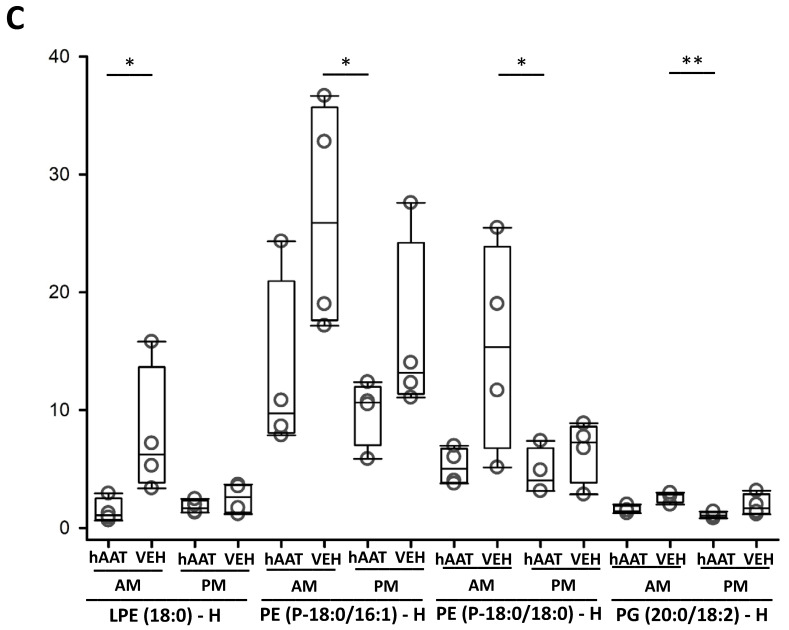
Lipidomics analysis of lipid classes enriched in urinary EVs from hAAT and vehicle treated db/db mice. Prior to lipid extraction, urinary EVs from each group was normalized to particles/mL. (**A**). Quantitative assessment of hexosylceramides (HCER). (**B**). Quantitative assessment of monoacylglycerol (MAG) and phosphatidylcholine (PC). (**C**). Quantitative assessment of lysophosphatidylethanolamine (LPE), phosphatidylethanolamine (PE), and phosphatidylglycerol (PG) from uEVs isolated from the urine during the active (AM) or inactive (PM) cycles of hypertensive diabetic mice treated with hAAT or vehicle. Samples were normalized to EV concentration. N = 4 hAAT AM, N = 4 vehicle AM, N = 4 hAAT PM, N = 4 VEH PM. AM reflects urine collected overnight during the active cycle of the mice and PM reflects urine collected during the day of the inactive cycle of the mice. * represents *p*-value < 0.05, ** represents *p*-value < 0.01.

To investigate whether the increase in EVs released and excreted into the urine by hAAT treated mice could be physiologically relevant and maybe contribute to the blood pressure lowering effect of hAAT, we extracted and analyzed lipids from uEVs of hAAT or vehicle treated diabetic db/db mice. As shown in Figure 11A, hAAT treatment resulted in the enrichment of hexosylceramides in uEVs and this was most evident in EVs isolated from urine samples collected during the animal’s active cycle. Our lipidomic analyses also showed other lipid classes were differentially enriched in the uEVs between the hAAT and vehicle treated groups after normalizing to EV count with sample buffer (Figure 11B,C).

## 4. Discussion

To our knowledge this is the first study investigating the potential role of hAAT as a treatment for salt-sensitive hypertension with diabetes as a co-morbidity. Here, we found that after a third dose of hAAT there was a significant decrease in systolic blood pressure in the hypertensive diabetic db/db mice. Our blood pressure data strongly supports our hypothesis that hAAT alleviates blood pressure in hypertensive diabetic db/db mice. In addition, immunohistochemistry showed a significant decrease in the protein expression of both ENaC alpha and MARCKS in the kidney of hAAT treated mice compared to the mice treated with vehicle. This suggests either hAAT is indirectly down-regulating ENaC through the reduction of MARCKS protein expression at the apical plasma membrane or directly inhibiting ENaC protein expression at the apical plasma membrane and presumably its proteolysis and activation through the rearrangement of the actin cytoskeleton or inhibition of various proteases that are responsible for cleaving and activating the channel.

Proteases play an important role in activating several epithelial transport proteins in the kidney that regulate blood pressure including ENaC. For example, proteases including furin [47], cathepsin B [21], cathepsin S [23], prostasin [48], and kallikrein [25] cleave ENaC subunits resulting in channel activation. Here, we investigated whether the protease inhibitor hAAT can lower blood pressure in the hypertensive diabetic kidney of diabetic db/db mice. We investigated the AAT dependent regulation of multiple epithelial transport mechanisms that are known to contribute indirectly or directly to blood pressure regulation. Since AAT expression, blood pressure regulation, and several proteins we investigated here are regulated by circadian rhythms, we collected samples and took physiological measurements during the inactive cycle and active cycles.

Some limitations of this study include not investigating how administering hAAT to hypertensive db/db mice affects renin production or hormones know to regulate ENaC nor did we investigate how prolonged treatment of hAAT affects the mice. The hAAT used in this study is clinical grade hAAT that is currently being used to treat human patients with alpha 1-antitrypsin deficiency. We are not aware of any toxicities in mice treated with hAAT. Future studies will focus on addressing these avenues of research.

Our data did not indicate a significant difference in cathepsin B or kallikrein activity in kidney lysates from hAAT treatment compared to vehicle-treated dbdb mice. This is not surprising because although we expected an increase in hAAT in the systemic circulation, and our data suggest hAAT enters kidney cells, it is not known if and how it enters distal tubules or collecting duct cells. Additionally, it is possible that hAAT decreases ENaC alpha and MARCKS protein density at the apical membrane through another mechanism that is yet to be elucidated.

There was no significant difference in electrolyte excretion between hAAT treated and vehicle treated diabetic db/db mice. This would suggest that hAAT decreases blood pressure in the diabetic db/db kidney through a mechanism that is independent of changes in sodium balance. The significant decrease in blood pressure that we have reported could be caused by systemic vasodilation. Further research is required to determine whether hAAT stimulates systemic vasodilation in salt sensitive hypertensive db/db mice.

In this study, we treated diabetic db/db mice with hAAT for less than one week before we investigated the effects on epithelial transport mechanisms in the kidney. Since other studies suggest hypertension exacerbates diabetic kidney disease and increases the risk of developing diabetic nephropathy [49,50,51], additional studies are necessary to determine whether chronic hAAT treatment can normalize blood pressure and mitigate poor outcomes that are associated with diabetes.

Urinary extracellular vesicles (uEVs) represent a rich source of biomarkers for disease pathogenesis. EVs, in general, are released from all cell types and they carry bioactive cargo including signaling lipids, metabolites, proteins and microRNAs that allow for intercellular communication, differentiation of cell types, and intracellular signaling. EVs present in the urine originate mainly from kidney and bladder epithelial cells since larger sized EVs are generally not filterable. The packaged cargo within EVs isolated from the conditioned media of kidney cells was previously shown to regulate ENaC in an in vitro model [52]. However, the packaged cargo enriched in uEVs is likely different in the pathophysiology of hypertension and diabetes. In this study we investigated whether hAAT treatment can induce EV release and excretion into the urine compared to vehicle treatment. These findings may be important to our understanding of the beneficial role of hAAT treatment in hypertension and diabetes since it could represent a mechanism for the removal of toxic or harmful cargo by EVs. It could also represent a mechanism for increased metabolism of lipids and proteins that accumulate during the pathophysiology of hypertension and diabetes.

Our lipidomics analysis showed HCER lipids were increased in the diabetic db/db mice treated with hAAT compared to vehicle. This intriguing finding may be involved in the mechanism of hAAT normalizing blood pressure in hypertensive diabetic mice. The de novo synthesis of CER was previously shown to be involved in the inflammatory response [53]. In another study, Dupre et al. investigated the role of ceramide metabolism in kidney injury [54]. This group found ceramides mediate cisplatin-induced acute kidney injury and the metabolism of these lipids is protective and helps to attenuate kidney injury [54]. Here, we show multiple HCERs including HCER (16:0), HCER (18:0), HCER (18:1, HCER (20:0), HCER (22:1), HCER (24:0), and HCER (24:1) are increased in urinary EVs released from hAAT treated hypertensive diabetic mice compared to vehicle treated control mice. Furthermore, the most significant increase in HCER production from CER metabolism was in urine samples collected at the beginning of the rest phase/cycle which reflects urine made during the active cycle of the mice. These results warrant further investigation to better understand how hAAT may be altering the lipid content of EVs and their release from kidney cells.

Taken together, the data presented here suggest hAAT treatment may be useful in the reduction of high blood pressure during the pathogenesis of diabetes. Additional studies are necessary to further investigate the mechanism of EV release, characterization of the packaged molecules within the EVs, and functional analysis of the role of these EVs in the hypertensive diabetic kidney after hAAT administration.

## Figures and Tables

**Table 1:** Urine Osmolality and Urine Electrolytes were measured using the urine collected during the treatment phase. N = 6 hAAT treated (3 females and 3 males) mice and N = 6 VEH (3 females and 3 males) mice. These results were analyzed using a One-Way ANOVA followed by a Holm–Sidak comparison.

		GROUP	MEAN + SEM	*p*-VALUE
**Na^+^** **(mmol/L)**	Active	AAT	156.167 ± 40.402	*p* = 0.793
		VEH	126.5 ± 29.567	
	Inactive	AAT	130.333 ± 19.736	
		VEH	158.667 ± 19.857	
**K+** **(MMOL/L)**	Active	AAT	21.483 ± 4.572	*p* = 0.861
		VEH	19.533 ± 4.636	
	Inactive	AAT	23.133 ± 5.223	
		VEH	24.983 ± 4.107999	
**CL+** **(MMOL/L)**	Active	AAT	177.667 ± 35.819	*p* = 0.953
		VEH	154.333 ± 30.81	
	Inactive	AAT	169.167 ± 33.893	
		VEH	176.167 ± 27.368	
**URINE OSMOLALITY** **(MOSM)**	Active	AAT	1268.16 ± 66.45	*p* = 0.947
		VEH	1295.5 ± 74.09	
	Inactive	AAT	1314.5 ± 90.74	
		VEH	1365.16 ± 193.31	

**Table 2:** Cathepsin S, Cathepsin B, and calpain protease activity in the plasma of db/db mice treated with hAAT or vehicle. Data is presented as mean ± SEM. AAT n = 6, VEH n = 6.

PROTEASE	GROUPS	MEAN ± SEM RELATIVE FLUORESCENCE UNITS (RFU)	STUDENT’S T-TEST COMPARISON	*p*-VALUE
**CATHEPSIN S**	AAT VEH	27,099.000 ± 4915.036 22,441.500 ± 3486.513	AAT vs. VEH	*p* = 0.457
**CATHEPSIN B**	AAT VEH	1972.333 ± 237.104 1829.833 ± 135.648	AAT vs. VEH	*p* = 0.613
**CALPAIN**	AAT VEH	38,126.667 ± 1198.951 38,614.167 ± 1524.387	AAT vs. VEH	*p* = 0.807

## Data Availability

All data are presented in the figures.
